# The SUMOylation machinery is dispensable for Tax-induced NF-kB activation

**DOI:** 10.1186/1742-4690-11-S1-O60

**Published:** 2014-01-07

**Authors:** Sabrina Pène, Amandine Bonnet, Laetitia Waast, Laurence Bénit, Claudine Pique

**Affiliations:** 1 INSERM, U1016, Institut Cochin, CNRS, UMR8104, Université Paris Descartes, Sorbonne Paris Cité, Paris, France

## 

The HTLV-1 Tax protein is a good example of the diversity and functional importance of post-translational modifications. Indeed, Tax is phosphorylated, acetylated, conjugated to at least K48- and K63-linked poly-ubiquitin chains as well as to SUMO-1 and SUMO-2/3 molecules. In previous studies, we and others proposed that these modifications regulate both the intracellular distribution of Tax and its ability to activate the NF-kB pathway. In particular, Tax SUMOylation was associated to the formation of Tax nuclear bodies believed to represent transcriptionally active structures. However, our recent finding that a Tax mutant intrinsically weakly SUMOylated remains able to fully activate a NF-kB promoter challenged the importance of SUMOylation and/or nuclear body formation in the NF-kB activity of Tax. In this study, we further explored the role of Tax SUMOylation by targeting Ubc9, the unique E2 SUMO conjugating enzyme. We found that either a catalytically inactive form of Ubc9 (Ubc9-C93S) or Ubc9 siRNA totally blocks Tax conjugation to endogenous SUMO-1 or SUMO-2/3, showing that as expected, Tax SUMOylation is under the control of the catalytic activity of Ubc9. We next observed that this absence of Tax SUMOylation prevents neither the activation of the cytoplasmic IKK complex nor the activation of a NF-kB promoter. Finally, we also observed that surprisingly, the ability of Tax to form nuclear body was also not affected by overexpression of Ubc9-C93S. These data provide the direct demonstration that Tax SUMOylation and nuclear body formation are dispensable for Tax-induced NF-kB activation.

